# Factors affecting willingness to share electronic health data among California consumers

**DOI:** 10.1186/s12910-017-0185-x

**Published:** 2017-04-04

**Authors:** Katherine K. Kim, Pamela Sankar, Machelle D. Wilson, Sarah C. Haynes

**Affiliations:** 1 University of California Davis, Betty Irene Moore School of Nursing, 2450 48th Street, Suite 2600, Sacramento, CA 95817 USA; 2 Department of Medical Ethics and Health Policy, University of Pennsylvania, 423 Guardian Drive, Blockley, 14, Philadelphia, PA19104-4884 USA; 3 Department of Public Health Sciences, Division of Biostatistics, Clinical and Translational Science Center, University of California Davis, 2921 Stockton Blvd, suite 1400, Sacramento, CA 95817 USA

**Keywords:** Consent, Ethics, Electronic health records, Health information exchange, Learning healthcare systems, Distributed research network

## Abstract

**Background:**

Robust technology infrastructure is needed to enable learning health care systems to improve quality, access, and cost. Such infrastructure relies on the trust and confidence of individuals to share their health data for healthcare and research. Few studies have addressed consumers’ views on electronic data sharing and fewer still have explored the dual purposes of healthcare and research together. The objective of the study is to explore factors that affect consumers’ willingness to share electronic health information for healthcare and research.

**Methods:**

This study involved a random-digit dial telephone survey of 800 adult Californians conducted in English and Spanish. Logistic regression was performed using backward selection to test for significant (*p*-value ≤ 0.05) associations of each explanatory variable with the outcome variable.

**Results:**

The odds of consent for electronic data sharing for healthcare decreased as Likert scale ratings for EHR impact on privacy worsened, odds ratio (OR) = 0.74, 95% CI [0.60, 0.90]; security, OR = 0.80, 95% CI [0.66, 0.98]; and quality, OR = 0.59, 95% CI [0.46–0.75]. The odds of consent for sharing for research was greater for those who think EHR will improve research quality, OR = 11.26, 95% CI [4.13, 30.73]; those who value research benefit over privacy OR = 2.72, 95% CI [1.55, 4.78]; and those who value control over research benefit OR = 0.49, 95% CI [0.26, 0.94].

**Conclusions:**

Consumers’ choices about electronically sharing health information are affected by their attitudes toward EHRs as well as beliefs about research benefit and individual control. Design of person-centered interventions utilizing electronically collected health information, and policies regarding data sharing should address these values of importance to people. Understanding of these perspectives is critical for leveraging health data to support learning health care systems.

## Background

Over the last decade, the expansion and improvement of electronic health records (EHRs) has become a national priority and is described as “foundational to achieve the nation’s health and wellness goals [1]”. To promote this goal the 2009 Health Information Technology for Economic and Clinical Health act (HITECH) allocated 35 billion dollars to incentivize the development and implementation of electronic health record (EHR) systems for all healthcare providers [2–4]. On many counts the effort has succeeded. By 2015 83.8% of hospitals had basic EHR systems, up from 15.6% in 2010, and physicians with a certified EHR system had increased from 30% to 75% [5].

HITECH’s goals however extend beyond improving the country’s fragmented EHRs. Nationwide adoption of EHRs is meant to enable development of a new model for healthcare, describes as a learning health care system (LHCS), in which patient information, captured at the point of care, is analyzed to assess treatment efficacy, safety, and quality of care, and fed back to improve patient care. The strategy of grounding research in patients’ usual care, intended to overcome the challenge biomedical research faces producing knowledge that transfers successfully from experimental protocol to daily life, has motivated many new approaches to research including pragmatic clinical trials, comparative effectiveness research (CER), and research on medical practices (ROMP) [6]. Each seeks to contribute to an LHCS model in different ways, but all share a defining feature, which is the need to multiply the uses of patient data. In addition to informing patient care, this data must now support quality improvement and research.

Few dispute the potential benefits of a LCHS model. However, implementing it presents more than a technical challenge. Transforming the patient encounter into research data also requires accepting changes to privacy and confidentiality, which are important foundations of patient trust and effective health care. Studies demonstrate broad support among patients and consumers for approaches to improving health care that rely on using patient information [7, 8]. They also have consistently found privacy to be a major concern [9–14]. The disjuncture between the technical demands of creating an information infrastructure to support LHCS models and respecting patient concerns about the privacy and control of their health information poses a key ethical challenge to this model of health care.

EHRs in themselves worry some patients who believe they offer fewer privacy protections than paper records [13]. A 2015 survey of a random sample of 1000 US residents suggests that this belief is waning, but still found 41.2% believed that EHRs compromised patient privacy [15]. Another recent study suggests that patients withhold more information from practitioners using EHRs than from those using paper records [16]. EHR privacy concerns have been confirmed even among subjects who agree that EHRs are more efficient than paper records [17] and are not necessarily limited to individuals who are less familiar with computers, [18] although other studies contradict this [12, 19]. Higher education has been associated with the belief that EHRs will improve quality of health care and security [15] but also with a decreased willingness to provide access to personal health information [20, 21].

The LHCS model requires EHRs that are fully integrated or linked into regional networks so that information about a patient’s care can be shared across the sites where it is delivered. Health information exchanges (HIEs) are one type of regional network that fulfills this need. To enter a patient’s identifiable health information into an HIE, the federal government recommends a “meaningful consent” process that includes patient education and information about how health information will be shared [22], but there is little evidence that this occurs in practice. Several studies on patient preferences have found that patients prefer an HIE opt-in model with explicit approval sought before sharing data [23–26]. However, providers may choose to offer an opt-out policy, in which patients must take active steps to prevent sharing of their health information [22]. Previous studies have also identified factors that influence willingness to share health information, such as poor health status, but results are contradictory [19, 27].

To realize the vision of a LHCS model patient data such as that collected through HIEs or otherwise aggregated through EHRs, also needs to be available for quality improvement and research. There is growing debate over whether and how patients should consent to this use of their health care information, often shared in de-identified form [6, 28, 29]. Some ethicists have argued that refusing to contribute to the public health in this way is shortsighted and that contributing one’s health care information should be seen as a social obligation, not a choice [1, 6, 28]. The fact that patient information is already routinely shared with researchers in de-identified form is not necessarily understood by patients [9, 30, 31] and studies about patient interactions with electronic health information systems do not always distinguish the different uses to which health data might be put [19].

Studies report broad support of the idea of using treatment-generated data to conduct research to improve medical care [32–34]. Respondents however also express concern that such research will jeopardize the privacy of their medical information [19, 27, 32–34], and that these concerns are sufficient to lead people to refuse to share health information or to strictly limit its use [33, 35].

There is a body of research examining attitudes that affect willingness to consent to participate in research studies. Many factors identified are associated with the protocol itself, such as side effects or the stringency and complexity of the protocol [36]. In addition to practical considerations, research participants also cite altruism [37–41] as influencing decisions to participate in research, which studies of willingness to participate in EHR-based research also cite [20, 27, 32].

Trust—in the health system, study staff, or a provider’s recommendation—has been shown to increase the likelihood that patients will participate in research [36, 37, 42–44], and distrust to decrease it [38, 39, 45, 46]. Sometimes, in EHR-based research, once a patients’ consent is obtained, the research proceeds without further contact or involvement with the patient. For some, this lack of connection signaled a lack of control, and created concerns such as for what future purposes their data might be used [33].

There is some evidence that patients’ willingness to share data for research may differ from willingness to share data for healthcare, although few studies have addressed both purposes together. A recent study on consent for data sharing in California found that patients are more likely to share de-identified health information for research purposes than to share identified information for healthcare purposes [47]. Minority identity has been shown to be linked with reduced willingness to share information for research purposes, [19, 48, 49] but one study linked Asian identity with a greater willingness to share for purposes of an HIE [26].

A better understanding of how consumer characteristics, attitudes, and beliefs affect willingness to consent to electronic data sharing may support the development of strategies to enhance the public’s trust in networks, and hence their participation in networked models that support the development of LHCS models to improve patient care. This study explores the factors that may affect a patient’s willingness to share electronic health data for both healthcare and research purposes.

## Objectives

The primary research questions addressed by the study were:

1. What are the factors associated with willingness to share health information electronically for healthcare?
2. What are the factors associated with willingness to share health information electronically for research?

## Methods

A random digit dialed, anonymous survey of California residents was conducted in both Spanish and English in early 2013. The survey was developed from themes that arose from patient focus groups and previously published surveys including items related to privacy, security, HIE, EHR, and health research and underwent cognitive testing before being fielded. The study was approved by the institutional review board at San Francisco State University. A detailed description of the study methodology and the complete survey is published elsewhere [47].

Two outcome variables were defined, one for HIE consent and one for research consent, each with four point scales (very likely, somewhat likely, somewhat unlikely, very unlikely) that were condensed to binary response categories of likely and unlikely. Health information exchange (HIE) was described in the survey as medical information shared electronically between the places where a patient receives medical care. HIE consent was addressed with the question “If you were offered the choice to have your medical information automatically shared electronically (without requiring any action by you) with the different places where you receive medical care, how likely would you be to agree to it?” The research consent question was “Some medical research studies use information that is already in electronic medical records. The information used for these studies is unidentified. This means the information does not include your name, address, or social security number. If medical researchers asked to use your unidentified medical information from your electronic medical record how likely or unlikely would you be to agree to that?

The explanatory variables were attitudes about quality, privacy and security, each of which had a five point responses (greatly improve, slightly improve, have no effect, slightly worsen, greatly worsen) that were condensed to three categories of improve, no effect, and worsen. The concepts of privacy and security were described as: Having a say in who can collect, use and share your medical information has to do with the privacy of your records. Having safeguards (including the use of technology) in place has to do with the security of your medical records. The three questions related to HIE attitudes were: “If doctors use electronic medical records, instead of paper records, how do you think that would affect the quality of medical care?” “Sometimes patients may need to share medical information with different places where they receive medical care. If medical information could be shared electronically between the places where a patient receives medical care, how do you think that would affect the privacy of medical information?” and “If medical information could be shared electronically between the places where a patient receives medical care, how do you think that would affect the security of medical records?”

Three questions addressed research attitudes. The first question, “How do you think that making unidentified data from electronic medical records available for research would affect the quality of medical research studies?” was similar to the question asked of HIE and also offered the same response categories of improve, no effect, and worsen. The second question which assessed the trade-off of the benefit of research over individual privacy was “How much do you agree or disagree with this statement: Research that could be beneficial to people’s health is more important than protecting people’s privacy.” The third question which assessed the trade-off of the value of individual control over research benefit was “How much do you agree or disagree with this statement: An individual’s right to control use of their medical information is more important than the possible benefits of medical research”.

Summary statistics were calculated for each variable – frequencies for the categorical variables and means and standard deviation for the continuous. Univariable tests were performed to assess the association of each explanatory variable with the binary outcome variable (likely or not likely to consent). Univariable logistic regressions were fit to estimate unadjusted odds ratios and their 95% confidence intervals. A logistic regression was performed using backward selection to test for significant associations of each explanatory variable with the outcome variable, while adjusting for the other explanatory variables. Nagelkerke’s generalized R squared was calculated to estimate the improvement over the null model for each multivariable logistic regression model. A p-value of .05 for a covariate in the final model was considered significant. All analyses were performed using SAS® software version 9.3.

## Results

There were 800 adult respondents with a response rate of 14.0%, a rate similar to recent national, random digit dialed surveys [50]. As previously reported, the survey respondents were more likely to be 65 years or older and college educated than the general California or US populations; less ethnically/racially diverse than the state, but more diverse than the US population. The California sample shows similar technology use characteristics to respondents in recent national Pew surveys [47]. Respondent characteristics are presented in Table 1. The mean age was 53 years (*m* = 53.0, *SD* = 17.29, range 18–99). The majority of respondents are likely to consent to sharing data for healthcare and research purposes. Table 2 provides a summary of attitude and consent outcome variables used in the following results.

**Table 1:** Sample characteristics

	Representativeness of sample			
Variable % (N)	Survey sample	95% CI	California^a^	US^a^
Total responses	100.0 (800)			
Gender				
Female	53.0 (424)	[49.5, 56.5]	50.3	50.8
Age, years				
18-64	72.0 (576)	[68.9, 75.1]	83.0	85.8
65 and older	25.0 (200)	[22.0, 28.0]	17.0	14.2
Race/Ethnicity				
White (not Hispanic/Latino)	56.0 (448)	[52.6, 59.4]	39.7	63.0
Hispanic/Latino	22.9 (183)	[20.0, 25.8]	38.1	16.9
Asian/Pacific Islander	8.0 (64)	[6.1, 9.9]	6.6	5.3
Black	4.6 (37)	[3.1, 6.1]	14.1	13.1
Mixed/other	4.9 (39)	[3.4, 6.4]	3.6	2.4
Native American	1.1 (9)	[0.4, 1.8]	1.7	1.2
Education				
Up to high school	23.9 (191)	[20.9, 26.9]		
Technical training/some college	27.6 (216)	[24.5, 30.7]		
College degree or higher	48.0 (384)	[44.5, 51.5]	30.3^b^	28.2^b^
Geography				
Urban	90.6 (673)	[88.6, 92.6]		
Veteran	11.8 (94)	[9.6, 14.0]	7.8	0.1
Income				
Median household income in (dollars) $	50,000-60,000		61,632	52,762
Online Technology Use (%)	Survey Sample	95% CI		US
Internet	86.9 (695)	[84.6, 89.2]		85^c^
email	95.1 (661)	[93.6, 96.6]		92^d^
Ever used email to contact your doctor or nurse	44.5 (294)	[41.1, 47.9]		
Used the internet to connect with other patients	13.2 (92)	[10.9, 15.5]		15.8^e^
Ever participated in an online patient community	10.9 (76)	[8.7, 13.1]		8^e^
Ever shared your own health information for a research project via online patient community (of those in online community)	9 (11.8)	[9.6, 14.0]		
Have an account for a personal health record	21.0 (168)	[18.2, 23.8]		

^a^2007-2011 American Community Survey 5-year Estimates. Available at https://www.census.gov/quickfacts/table/PST045216/00, accessed 8/15/2013

^b^Population 25 years of older

^c^ http://www.pewinternet.org/fact-sheet/internet-broadband/ Spring 2013 Survey

^d^ http://www.pewinternet.org/Reports/2011/Search-and-email/Report.aspx 2011 data

^e^ http://www.pewinternet.org/2013/01/15/health-online-2013/

**Table 2:** Summary of attitude and consent variables

	Percentage		
Attitudes	Improve	No effect	Worsen
EHR effect on medical quality	73.5	15.0	6.8
EHR effect on privacy	22.0	25.6	52.4
EHR effect on security	37.0	20.3	42.7
HIE effect on privacy	28.9	30.7	40.3
HIE effect on security	30.6	27.0	42.5
EHR effect on research quality	74.3	12.8	8.0
Values	Agree	Disagree	
Research benefit over privacy	50.6	46.8	
Control over research benefit	69.8	26.9	
Consent	Likely	Unlikely	
Share for healthcare (HIE)	56.4	42.0	
Share for research	74.8	23.4	

### Research question 1. What are the factors associated with willingness to electronically share health information for healthcare?

For the univariable tests, health status and all privacy and security attitudes were significant at the .05 level, while race was significant at the .10 level. See Table 3. No other covariates were significant.

**Table 3:** Unadjusted relationships between characteristics and attitudes and likelihood of HIE consent

Characteristics	N	Odds ratio	95% confidence interval	*P*
Gender: Female vs. Male	784	0.88	(0.67, 1.20)	0.39
Race	767			0.08^b^
Hispanic/Latino		1.45	(1.01, 2.08)	
Black		0.65	(0.33, 1.27)	
Asian/Pacific Islander		0.95	(0.56, 1.62)	
Other		0.73	(0.40, 1.33)	
White, not Hispanic/Latino (ref)		--	--	
Income	619			0.11
Less than $40,000		1.40	(0.95, 2.00)	
$40 K to < $80 K		1.50	(0.97, 2.20)	
$80 k or greater (ref)		--	--	
Education	778			0.21
Up to High School Diploma		1.50	(0.80, 2.90)	
HS diploma to Bachelor’s Degree		0.88	(0.65, 1.20)	
More than a Bachelor’s Degree (ref)		--	--	
Geography: Urban vs. Rural	732	1.30	(0.79, 2.20)	0.29
Health Status	781			0.02^a^
Very good		0.66	(0.49, 0.89)	
Fair		0.57	(0.29, 1.10)	
Poor (ref)		--	--	
Has regular provider (yes vs. no)	779	1.10	(0.78, 1.40)	0.72
Has used email to contact provider (yes vs. no)	654	0.87	(0.64, 1.20)	0.39
Currently has personal health record (yes vs. no)	774	0.92	(0.65, 1.30)	0.64
Attitudes (Likert scale)				
EHR effect on medical quality	752	0.59	(0.51, 0.69)	<0.001^a^
EHR effect on privacy	730	0.66	(0.58, 0.74)	<0.001^a^
HIE effect on privacy	752	0.69	(0.61, 0.78)	<0.001^a^
EHR effect on security	751	0.71	(0.64, 0.80)	<0.001^a^
HIE effect on security	741	0.66	(0.59, 0.75)	<0.001^a^

^a^Significant at the .05 level

^b^Significant at the .10 level

For the logistic regression, the backward selection procedure eliminated the covariates in the following order with the following p-values: race (*p =* 0.93), urban (*p =* 0.64), personal health record (PHR) (*p =* 0.61), gender (*p =* 0.58), EHR effect on security (*p =* 0.58), regular provider (*p =* 0.40), health status (*p =* 0.40), EHR effect on privacy (*p =* 0.27), age (*p =* 0.18), and income (*p =* 0.10). After controlling for the other included variables, the following covariates were significant: education (*p =* 0.013), ever emailed provider (*p =* 0.001), EHR effect on medical quality (*p <* 0.001), HIE effect on privacy (*p =* 0.003), HIE effect on security (*p =* 0.028).

The odds of consenting to HIE were lower for those with low education levels compared to high, i.e., high school compared to college graduate OR 0.52, 95% CI [0.11, 2.43] and some college vs. college graduate, OR 0.51, 95% CI [0.33, 0.80]. The odds for those who had ever emailed their provider were only 47% of those who had not, 95% CI [0.30, 0.74], or equivalently, the odds for those who had not ever emailed their provider were almost twice that of those who had. Attitudes also affect HIE consent. For every unit increase in the Likert scale response for EHR effect on medical quality, i.e. from improve to no effect to worsen, the odds of consent decreased by a factor of 0.59. For every unit increase in HIE effect on privacy, the odds of consent decreased by a factor of 0.74. And for every unit increase in HIE effect on security, the odds of consent decreased by a factor of 0.80. (Table 4). Nagelkerke’s adjusted R-squared was 0.13, which indicates about a 13% improvement in model prediction over the null model of no association with any of the covariates, i.e., simply using the observed proportions.

**Table 4:** Adjusted odds ratios for effects on likelihood of HIE consent

	Odds ratio estimates		
Effect	Point estimate	95% Wald confidence limits	
Education			
Up to High School diploma	0.52	0.11	2.43
HS diploma to Bachelor’s Degree	0.51	0.33	0.80
More than a Bachelor’s Degree (ref)	--		
Has PHR (yes vs no)	0.47	0.30	0.74
EHR effect on medical quality (Likert from improve to worsen)	0.59	0.46	0.75
EHR effect on privacy (Likert from improve to worsen)	0.74	0.60	0.90
EHR effect on security (Likert from improve to worsen)	0.80	0.66	0.98

### Research question 2: What are the factors associated with willingness to electronically share health information for research?

For the univariable tests, race/ethnicity, income, education, having a regular provider, emailing provider, and PHR characteristics were significant at the .05 level. The three research attitudes were also significant. See Table 5.

**Table 5:** Unadjusted relationships between characteristics and attitudes and likelihood of research consent

Characteristics	N	Odds ratio	95% confidence interval	*P*
Gender: Female vs Male	782	0.77	(0.55, 1.10)	0.11
Age (years)	763	1.01	(0.99, 1.02)	
Race/Ethnicity	766			<0.001^a^
Hispanic/Latino		0.44	(0.30, 0.66)	
Black		0.35	(0.17, 0.71)	
Asian/Pacific Islander		0.52	(0.29, 0.94)	
Other		0.72	(0.35, 1.48)	
White, not Hispanic/Latino (ref)		--	--	
Income	620			0.048^a^
Less than $40,000		--	--	
$40 K to < $80 K		0.55	(0.33, 0.91)	
$80 k or greater (ref)		0.80	(0.34, 0.68)	
Education	772			<0.001^a^
Up to High School diploma		0.34	(0.14, 0.66)	
HS diploma to Bachelor’s Degree		0.48	(0.34, 0.68)	
More than a Bachelor’s Degree (ref)		--	--	
Geography: Urban vs Rural	731	1.36	(0.77, 2.38)	0.29
Health status	778			0.66
Very good		1.38	(0.68, 2.81)	
Fair		1.38	(0.67, 2.83)	
Poor (ref)		--		
Have regular provider (yes vs no)	777	2.03	(1.44, 2.85)	<0.001^a^
Ever emailed provider (yes vs no)	655	1.92	(1.29, 2.85)	0.001^a^
Have PHR (yes vs no)	772	2.25	(1.40, 3.62)	<0.001
Attitudes				
EHR effect on research quality (Likert)	749			<0.001^a^
Improve		11.68	(6.56, 20.80)	
No effect		1.99	(1.02, 3.85)	
Worsen (ref)		--	--	
Research benefit over privacy (agree vs disagree)	765	1.65	(1.18, 2.31)	0.003^a^
Control over research benefit (agree vs disagree)	759	0.61	(0.41, 0.92)	0.02^a^

^a^Significant at the .05 level

For the logistic regression, the backward selection procedure eliminated the covariates in the following order with their respective p-values: gender (*p =* 0.75), health status (*p =* 0.65), regular provider (*p =* 0.48), ever emailed provider (*p =* 0.31), geography (*p =* 0.15), income (*p =* 0.13), and age (*p =* 0.11). After controlling for the other included variables, the following covariates were significant: race (*p =* 0.01), education (*p =* 0.01), PHR (*p =* 0.05), EHR effect on research quality (*p <* .0001), research benefit over privacy (*p =* 0.0005), and control over research benefit (*p =* 0.03). (Table 5). Nagelkerke’s adjusted R-squared was 0.17, indicating a 17% improvement over the null model.

The multivariable regression results are shown in Table 6. The odds of individuals of all race/ethnic groups (Hispanic, black, Asian and other) consenting to research data sharing were lower compared to white, OR = 0.37, 0.83, 0.28, and 0.58 respectively. The odds of consent for lower education levels are smaller compared to higher levels, i.e. high school vs. college grad, OR = 0.80, 95% CI [0.16, 4.05]; and some college vs. college grad OR = 0.42, 95% CI [0.25, 0.73]. The odds of consent for those with a PHR are twice that of those who don’t have PHR, OR = 1.99, 95% CI [1.01, 3.92].

**Table 6:** Adjusted odds ratios for effects on likelihood of sharing for research

	Odds ratio estimates		
Effect	Point estimate	95% Wald confidence limits	
Race/Ethnicity			
Hispanic/Latino	0.37	0.20	0.71
Black	0.83	0.25	2.77
Asian/Pacific Islander	0.28	0.12	0.67
Other	0.58	0.20	1.72
White, not Hispanic/Latino (ref)	--		
Education			
Up to High School Diploma	0.80	0.16	4.05
HS diploma to Bachelor’s Degree	0.42	0.25	0.73
More than a Bachelor’s Degree (ref)	--		
Has EMR (yes vs no)	1.99	1.01	3.92
Sharing affects research			
Improve	11.26	4.13	30.73
Neutral	1.74	0.57	5.29
Worsen (ref)	--		
Research benefits more important that privacy (agree vs disagree)	2.72	1.55	4.78
Individual rights more important than benefits of research (agree vs disagree)	0.49	0.26	0.94

The odds ratio of consent for those who think EHR will improve research quality compared to those who think it will worsen is 11.26, 95% CI [4.13, 30.73] while the odds ratio for those who are neutral is 1.74 compared to those who think it will worsen quality, 95% CI [0.57, 5.29]. The odds of those who value research benefit over privacy are 2.72 times those who don’t, 95% CI [1.55, 4.78] while the odds of those who value individual control over research benefit are 0.49 those who don’t, 95% CI [0.26, 0.94]. Nagelkerke’s adjusted R squared was 0.26, indicating about a 26% improvement over the null model.

## Discussion

### Demographic factors

While we did not find an association between ethnic or racial identity and willingness to consent to electronic sharing of health information for healthcare, we did find a significant association with respect to sharing for research. This is in line with other studies that have shown that minority identity was linked with reduced willingness to share information (although not specifically electronic information) for research purposes [19, 48, 49] and Asian identity linked with a greater willingness to share for purposes of HIE [26]. However, in the context of consent to participate in research, a previous systematic review that found no difference in consent rates between minority--African Americans, Hispanics-- and non-Hispanic whites [51]. While we did not find health status to be a predictor, other studies have found that patients with poorer health are more likely to consent to a research study [52, 53].

### Experience with technology

The mere presence of experience with technology for consumers is not indicative of acceptance of electronic sharing of health information. For example, those who used email to contact providers had lower odds of consenting to HIE but those who used a PHR had greater odds of consenting to research exchange. There may be unintended consequences of greater use of technology such as HIE including lack of confidence among patients that appropriate safeguards are in place to protect the privacy and security of their health information [54]. Deeper exploration of past health technology experience may elucidate these relationships.

### Attitudes about EHR and HIE

Attitudes related to EHR and HIE do affect willingness to consent to electronic data sharing for both healthcare and research. For healthcare, those who believe that sharing healthcare data through HIEs improves privacy and safety are more likely to consent to share data for healthcare purposes. Those who believe EHR positively impacts healthcare quality and research quality are more likely to consent to electronic data sharing for both research and healthcare. This association between EHR and research quality is noteworthy with an odds ratio of 11.26. A recent small study of consumers in New York found a similarly high level of support for sharing healthcare information through HIEs particularly among those who believed HIE would improve privacy and security of medical records [55].

Tierney et al. found that patients often prefer restricting provider access to their EHR data; and providers accessed EHR for those patients who had requested restricted access far less often when they knew the patient’s preference [56]. An area of future inquiry would be whether and how provider communication regarding their own beliefs about electronic data sharing, use of EHRs, privacy and security affects patients’ willingness and subsequent participation in electronic data sharing.

In addition, tradeoffs between values related to societal benefits of research, individual control, and privacy impact willingness to consent in research. Individuals may make trade-offs when considering whether to share their health information for research.

These findings taken together suggest that attitudes about information technology might be more complex than previous research has recognized. Our findings add new depth of understanding that attitudes about technology’s role in healthcare and research are important dimensions in individuals’ willingness to participate in electronic data sharing. Simplistic characterizations of personal preferences based on demographic factors or previous technology experience may not be adequate for designing person-centered networks. They add a new dimension to questions concerning trust and research participation, particularly the need to examine the interaction between the electronic basis of LHCS research, trust, and willingness to participate. They also suggest a need to examine the basis of attitudes toward electronic data sharing, and in particular to disambiguate the influence of experience with computer technology generally (for example in banking or shopping) and experience specific to health care. This information might help to inform strategies to address patient concerns about LHCS research demands. This need is particularly critical in light of ethics proposals to reframe this type of research participation as obligatory rather than voluntary [28].

The logistic regression models for factors affecting willingness to share electronic data indicated modest improvement in explanation of variability over the null model (R-squared of 13% for healthcare and 17% and 26% for research). There is much room for future research to deepen our understanding of the role of values and beliefs in decisions about electronic health information sharing.

### Limitations

This study was designed as a statewide survey to help inform California policy making and the stakeholder-informed design of the Scalable National Network for Effectiveness Research distributed research network (DRN) [11]. Although the demographics of the survey sample are similar to the general population there are important differences which may limit the generalizability of the findings to either California or the US. The sample is older and has a relatively high education level compared to the general public. Thus respondents may have different experiences related to healthcare policy and technology which lead to varying attitudes. Another limitation is the response rate of 14%. Although comparable to other random-digit dial national surveys, the low response rate tempers inferences made from the results. In order to better understand the relationship of attitudes and experience with EHRs/HIEs and consent behavior, prospective or natural experiments would be illuminating.

## Conclusions

Learning healthcare systems inextricably link the domains of practice and research. They depend on electronically networked partners, including the person and their caregivers, in order to collect relevant data, analyze information, and understand how to move health system levers that lead to improvements in health in alignment with each person’s needs, values, and preference which is the hallmark of person-centeredness in healthcare. Consequently, understanding of the intricacies of individuals’ attitudes about electronic data sharing, whether those data were collected for healthcare purposes, or are being reused for research, is critical to developing networked systems that embody person-centeredness.

## Abbreviations

DRN: Distributed research network
EHR: Electronic health record
HIE: Health information exchange
HITECH: 2009 Health Information Technology for Economic and Clinical Health act
LHCS: Learning healthcare system
PHR: Personal health record
